# Morphological Variation in Bumblebees (*Bombus terrestris*) (Hymenoptera: *Apidae*) After Three Decades of an Island Invasion

**DOI:** 10.1093/jisesa/iead006

**Published:** 2023-02-28

**Authors:** Cecilia Kardum Hjort, Henrik G Smith, Andrew P Allen, Rachael Y Dudaniec

**Affiliations:** Department of Biology, Lund University, Lund, SE-223 62, Sweden; Department of Biology, Lund University, Lund, SE-223 62, Sweden; Centre for Environmental and Climate Science, Lund University, Lund, SE-223 62, Sweden; School of Natural Sciences, Macquarie University, Sydney, 2109, NSW, Australia; School of Natural Sciences, Macquarie University, Sydney, 2109, NSW, Australia

**Keywords:** *Bombus terrestris*, bumblebee, environmental variation, invasion, morphology

## Abstract

Introduced social insects can be highly invasive outside of their native range. Around the world, the introduction and establishment of the eusocial bumblebee *Bombus terrestris* (L. 1758) (Hymenoptera: Apidae) has negatively impacted native pollinators and ecosystems. Understanding how morphological variation is linked to environmental variation across invasive ranges can indicate how rapidly species may be diverging or adapting across novel ranges and may assist with predicting future establishment and spread. Here we investigate whether *B. terrestris* shows morphological variation related to environmental variation across the island of Tasmania (Australia) where it was introduced three decades ago. We collected 169 workers from 16 sites across Tasmania and related relative abundance and morphology to landscape-wide climate, land use, and vegetation structure. We found weak morphological divergence related to environmental conditions across Tasmania. Body size of *B. terrestris* was positively associated with the percentage of urban land cover, a relationship largely driven by a single site, possibly reflecting high resource availability in urban areas. Proboscis length showed a significant negative relationship with the percentage of pasture. Wing loading and local abundance were not related to the environmental conditions within sites. Our results reflect the highly adaptable nature of *B. terrestris* and its ability to thrive in different environments, which may have facilitated the bumblebee’s successful invasion across Tasmania.

## Graphical Abstract

**Graphical Abstract:**
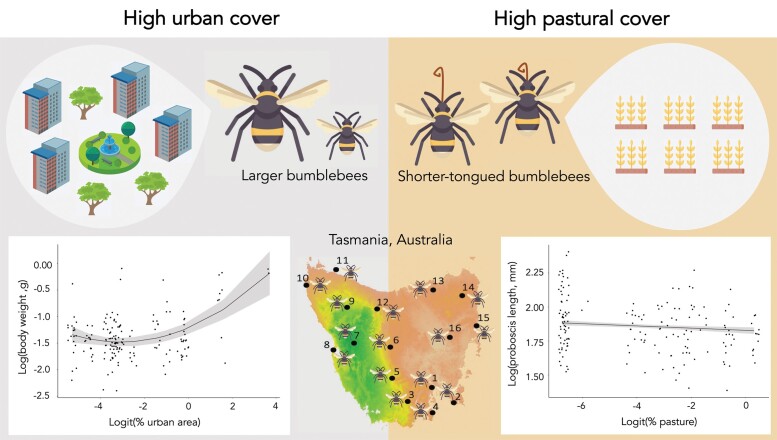

Introductions of species outside of their native ranges can drastically affect community structure and alter ecosystem processes (Mack et al. 2000, Morales et al. 2017). Insects are one of the most common groups of terrestrial invaders (Bradshaw et al. 2016) and social and eusocial Hymenoptera (wasps, ants, and bees) are particularly invasive worldwide (Russo 2016, Manfredini et al. 2019, Ghisbain et al. 2021).

Understanding the biological processes that facilitate insect invasions is important for analyzing the risk of species establishing in novel environments (Finch et al. 2021). The interplay among morphological variation, dispersal, and survival, whether it be via plasticity or evolution, is known to facilitate the rapid spread of invasive or pest insects (e.g., Yadav et al. 2018, Common et al. 2020). In insects, traits related to dispersal, such as wing morphology (Greenleaf et al. 2007, Hernández-L. et al. 2010, Renault 2020), wing muscle size (Therry et al. 2014), flight speed (Lombaert et al. 2014), and body size (Hemptinne et al. 2012, Laparie et al. 2013), are particularly relevant and may facilitate the expansion of invasive species.

In social bees, traits related to dispersal and foraging are likely to be important for adapting to shifting environmental conditions during invasion. Social bees also demonstrate large intra-specific morphological variation in body size. Because body size varies within colonies (up to 10-fold) and across locations, it is considered to be a plastic trait (Chole et al. 2019) that is responsive to different environmental conditions (Theodorou et al. 2020). Body size in social bees is also affected by variation in growth conditions during ontogeny (Schmid-Hempel et al. 2007, Rotheray et al. 2017, Gerard et al. 2018, Malfi et al. 2019, Guiraud et al. 2021). Body size may indirectly affect competition between invasive and native bees if it is related to key fitness components, as suggested by Inoue (2011) who observed selection for larger body size in invasive *Bombus terrestris* (L. 1758) (Hymenoptera: Apidea) 20 yr after its invasion to Japan. Proboscis length in bees has instead been related to the morphological characteristics of the flowers used in foraging (Inouye 1980, Harder 1985, Inoue and Yokoyama 2006, Klumpers et al. 2019), suggesting a genetically adaptive response to variation in local floral resources (Miller-Struttmann et al. 2015).

Among the eusocial bees, the European buff-tailed bumblebee, *B. terrestris,* is expanding worldwide (Dafni et al. 2010), both due to accidental and deliberate introductions (Russo 2016). Morphological variation in *B. terrestris* workers across native environments is well documented, such as larger body size associated with urban land use (Theodorou et al. 2020) and with higher availability of forage (Persson and Smith 2011, Tommasi et al. 2022). Even though *B. terrestris* is a habitat generalist (Dafni et al. 2010, Mossberg and Cederberg 2012), the abundance of workers of native *B. terrestris* has been found to vary with environmental conditions, likely driven by the availability of floral resources (Westphal et al. 2003, Heard et al. 2007, Timberlake et al. 2021).

One of the most rapid and successful cases of *B. terrestris* establishment outside of its native range is in the Australian island state of Tasmania, where bumblebees were first discovered in 1992 (Semmens et al. 1993, Hingston 2006a). The initial colonization event is thought to have involved only a few (perhaps as low as three) colonizing *B. terrestris* queens originating from New Zealand (Schmid-Hempel et al. 2007). Within a decade, it had spread from the city of Hobart where it was introduced to almost the entire island (Hingston et al. 2002). The Tasmanian population exhibits very low genetic diversity compared to native populations in Europe and to introduced populations in New Zealand (Schmid-Hempel et al. 2007). It is believed to be inbred (Hingston 2005), but does not show signs of inbreeding depression (Schmid-Hempel et al. 2007). This may be related to the haplodiploid sex determination system of bees, that results in increased purging of deleterious recessive alleles expressed in haploid males (Schmid-Hempel et al. 2007). Tasmanian *B. terrestris* may also further benefit from a reduced load of pathogenic viruses (Allen et al. 2007), although two viruses, the Kashmir bee virus (KBV) and Sacbrood virus (SBV) are now prevalent in the south-eastern Tasmanian populations (Fung et al. 2018).

The widespread invasion of *B. terrestris* has negatively impacted Tasmanian ecosystems, because it competes with and displaces native bees and other pollinators (e.g., Hingston and McQuillan 1999, Hingston and Wotherspoon 2017), in turn reducing the pollination efficiency of native plants (i.e., due to nectar robbing or physical damage to flowers) (Hingston and McQuillan 1998a). Pollination from *B. terrestris* is further thought to facilitate faster spread of invasive weeds in Tasmania by increasing their seed production (Hingston 2006b). Mainland Australia is currently free of *B. terrestris*, but remains at risk due to the species’ high vagility (Hingston 2007, Fijen 2021) and the availability of suitable habitats and appropriate climates, similar to those found in Tasmania (Acosta et al. 2016).

Here we examine whether *B. terrestris* exhibits significant variation in morphology and abundance related to environmental variables across Tasmania. Environmental conditions vary widely across Tasmania, with large differences in temperature and precipitation between the east and west of the island (Hill et al. 2009), as well as large differences in land use and elevation (Zhai et al. 2011). These heterogeneous environmental conditions have potential to drive morphological shifts in invasive *B. terrestris*. The nature and magnitude of these morphological shifts are relevant for understanding how rapidly invasive species respond and adapt to shifting environmental conditions. Further this study aims to yield insight into how *B. terrestris* might respond to similar environments beyond its current invasive range, including the Australian mainland.

## Methods

### Study Design and Sampling

**Fig. 1.**
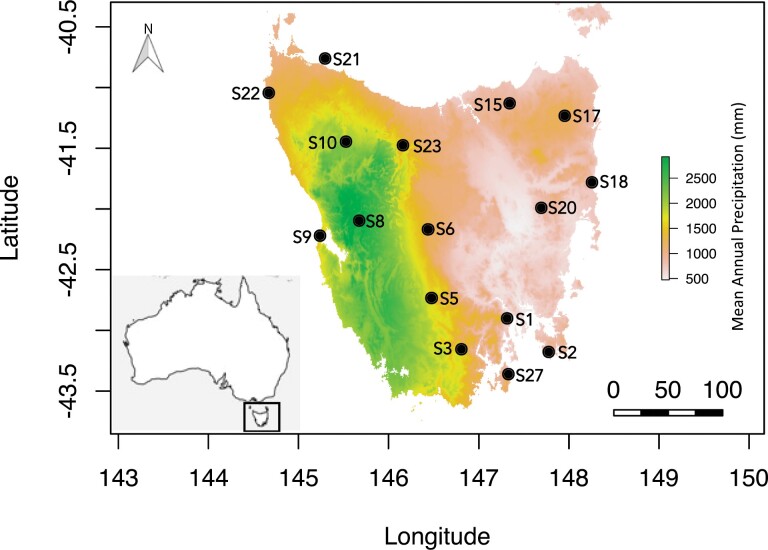
Sample sites of *B. terrestris* (*n* = 16) are shown (see Table 1 for corresponding site names) over mean annual precipitation (mm) across Tasmania, within Australia (inset). Scale bar is in kilometers.

We sampled *B. terrestris* workers at 16 sites across the Australian island state of Tasmania using previous records of the species’ occurrence to inform site selection (Hingston et al. 2002, Hingston 2006a). The sites encompass the two climatic zones of Tasmania that split the wetter western and the drier eastern sides (Fig. 1), covering a wide range of conditions in terms of climate, topography, land use (urban, pasture, forest, woodland), vegetation cover, and vegetation height (Fig. 1, Supp Fig. 1–2, Supplementary Material 1 [online only], hereafter ‘A1’). Each site was located a minimum of 37 km apart (mean: 52 km, ±s.e. 3.35 km). Bumblebees were generally caught in open areas, where flowers were present (e.g., along road verges and in patches of grass with flowers) in both residential and rural/wild areas, in meadows, adjacent to beaches, forest margins in rural areas, and gardens and parks.

**Table 1.** Summary of the three final morphological and seven environmental variables analyzed for each site (Site name, Site ID). The number of individuals from each site included in the analyses is presented as *N*. The morphological variables are presented as mean with standard error (s.d.). Total number of captured bumblebees per site and total search time (min) per site are also presented

Site ID	Site name	*N*	Total time (min)	# bees caught/site	Morphological variables			Environmental variables						
					Body weight (g)	Proboscis length (mm)	Wing loading (g/cm^2^)	Mean AnnualTemp (C°)	Mean AnnualPrecip (mm)	Season Precip (mm)	% pasture	% urban area	Veg. height (m)	AvgWind Summer (m/s)
1	Hobart	10	270	21	0.63 ± 0.27	8.34 ± 01.76	0.36 ± 0.10	11.74	709.88	17.17	0	86.46	32.28	5.81
2	Port Arthur	13	351	33	0.24 ± 0.10	6.89 ± 0.96	0.26 ± 0.05	10.85	891.16	14.55	1.18	0.17	43.23	5.76
3	Hartz MTN	10	75	19	0.34 ± 0.29	6.80 ± 1.46	0.34 ± 0.09	9.82	1360.45	22.58	10.08	3.56	44.29	5.63
27	Bruny Island	10	14	11	0.28 ± 0.06	6.70 ± 1.06	0.18 ± 0.04	11.39	838.75	13.29	11.06	18.54	25.23	6.19
5	South West NP	10	75	21	0.31 ± 0.22	6.26 ± 1.16	0.28 ± 0.06	9.68	1212.35	23.26	6.25	26.25	28.91	4.79
6	Lake St Clair	11	30	23	0.28 ± 0.08	6.73 ± 0.60	0.24 ± 0.04	9.68	1223.70	23.47	0	0	22.41	3.91
8	Franklin-Gordon WR NP	12	60	16	0.24 ± 0.04	6.41 ± 0.93	0.19 ± 0.05	10.68	2613.82	23.90	0	0.58	23.58	5.32
9	Macquarie Heads	11	75	24	0.22 ± 0.05	6.11 ± 0.51	0.27 ± 0.03	11.96	1467.57	27.46	0	1.94	26.32	6.42
10	Tikkawoppa/Waratah	12	75	16	0.23 ± 0.03	6.15 ± 0.41	0.28 ± 0.02	8.91	2041.05	35.94	1.38	45.34	27.92	4.91
22	Arthur River	9	90	9	0.22 ± 0.06	6.17 ± 0.54	0.18 ± 0.04	12.57	1167.80	35.39	0	7.39	37.91	6.42
21	Stanley	9	75	10	0.27 ± 0.07	6.39 ± 0.63	0.28 ± 0.01	13.02	1039.70	34.41	34.88	41.93	18.79	6.12
23	Cethana	9	60	20	0.28 ± 0.05	6.49 ± 0.76	0.35 ± 0.28	9.83	1477.95	32.83	2.06	3.77	37.52	4.62
15	Nabowla	10	90	19	0.25 ± 0.09	6.18 ± 0.63	0.23 ± 0.07	12.49	873.60	28.89	10.21	1.93	18.74	4.72
17	Rayers Hill (Weldborough)	11	150	19	0.20 ± 0.04	6.06 ± 0.57	0.23 ± 0.08	10.17	1169.31	24.61	5.74	3.16	31.80	4.23
18	Douglas-Apsley	10	90	21	0.25 ± 0.05	6.02 ± 0.71	0.39 ± 0.22	12.63	691.63	19.45	35.13	2.5	17.43	5.23
20	Campbell Town	12	60	21	0.24 ± 0.07	6.21 ± 0.61	0.28 ± 0.01	9.61	759.57	16.58	57.67	2.84	14.05	4.45

At each site, free-flying workers were opportunistically sampled during the active summer flight period in February 2020. Three people searched for and captured *B. terrestris* workers using handheld entomological sweep nets with the aim of capturing a minimum of 10 individuals (except for site 10, where sampling was interrupted after 9 individuals were captured). We stopped sampling after 90 min even if a total of 10 individuals had not been caught (Table 1). Collected workers were placed in 5 ml plastic tubes that were kept in cooling-boxes (5°C) to induce chill coma (loss of flight muscle function, MacMillan and Sinclair 2011). They were subsequently euthanized in a freezer (−20°C) for approximately 3 hr before preservation in 70% ethanol. We recorded the central coordinates of each site using Google Maps Satellite.

### Morphological Variables

Out of the total number of captured workers (Table 1), we randomly chose 10–15 individuals per site for which we measured a suite of morphological variables. In the lab, each bumblebee was blotted on tissue paper and left to dry for 5 min at room temperature to allow ethanol to evaporate, and then weighed (0.001 g precision) with a fine scale. Inter-tegular distance (ITD, 0.01 mm precision) was measured for each bumblebee using a digital caliper. Bumblebees were placed ventrally on millimeter paper and photographed using a digital camera microscope (Veho DX-2 USB 5MP Microscope). Subsequently, each bumblebee had its proboscis and one wing-pair (i.e., the most intact small and large wing, respectively) carefully dissected and placed on millimeter-paper and photographed under the camera microscope. Body length (mm), proboscis length (mm) (prementum and glossa), wing length (mm), and wing area (mm^2^) measurements were measured from photographic images at 0.01 mm precision using ImageJ v1.5.3 (Schneider et al. 2012). To characterize wing morphology while helping to control for body size variation among *B. terrestris* workers, we calculated wing loading as the body weight in grams divided by the total wing area in cm^2^ (Supp Table 1, Supplementary Material 2 [online only], hereafter ‘A2’). For proboscis length, we controlled for body size statistically as described below. Due to missing data (i.e., due to damaged proboscis or wings that could not be measured), the final number of workers used for analysis was 169 (*N* = 9–13 per site, Supp Table 1, [online only], A2).

### Morphology Variable Correlations

The seven morphological variables (body weight, ITD, body length, proboscis length, wing length, and area and wing loading) were first transformed as the natural logarithm, to linearize trends and stabilize variances. Interrelations among morphological variables were examined using Pearson correlation in R v.1.2.5 (R Core Team 2022). Because correlations among morphological variables were generally high (*r* > 0.70) (Supp Table 1 [online only], A1) and likely driven by body size differences, we focussed on a restricted number of relatively independent variables. Because body weight and ITD were highly correlated (*r* = 0.79), and ITD is often used as a proxy for body weight (Cane 1987, Grula et al. 2021), we only kept body weight for further analyses. Proboscis length was correlated with body weight (*r* = 0.78, Supp Table 1 [online only], A1), but we accounted for this in the statistical analyses by using body weight as a covariate. We also kept wing loading as an estimate of flight capacity (mobility). To assess inter- versus intra-site variation in bee morphology, we calculated the intra-class correlation coefficient as the site-level repeatability (*R*) for each of the three dependent variables using the R-package rptR (Nakagawa and Schielzeth 2010). When estimating site-level repeatability for proboscis length, we controlled for body size by including it as a fixed effect. Significance for *R* was assessed using a likelihood ratio test, and a 95% confidence interval (CI) for *R* was calculated based on 1,000 bootstrapping runs.

### Environmental Variables

We considered environmental predictors related to climate, elevation, land cover, vegetation cover, and vegetation height. We used the geographic coordinates for each sampling site to extract the following variables from WorldClim v2.1 at 1 km^2^ resolution averaged across the years 1970–2000 (Fick and Hijmans 2017), which have been found to be biologically meaningful for the distribution and climatic niche of *B. terrestris* and other bumblebee species (Rasmont et al. 2015, Penado et al. 2016): mean annual temperature (hereafter ‘MeanAnnualTemp’ in °C), mean annual precipitation (hereafter ‘MeanAnnualPrecip’ in mm), temperature seasonality (hereafter ‘SeasonTemp’, the difference between the hottest and coldest month in °C) and precipitation seasonality (hereafter ‘SeasonPrecip’, the difference between the wettest and driest month in mm). We also extracted and averaged values for December to February of the monthly variables of mean annual wind speed (m s^−1^), maximum, minimum, and average temperature (°C) (hereafter ‘AvgSummerWind’, AvgSTempMax’, ‘AvgSTempMin’, ‘AvgSTempMean’), as summer is the peak of the active flying period of *B. terrestris*. Wind speed has been found to negatively affect bumblebee foraging activity (Sanderson et al. 2015) and maximum and minimum temperatures are relevant for bumblebee distributions. Elevation was considered since bumblebees present at higher elevations may show adaptation to colder temperatures and reduced air density (Geue and Thomassen 2020, Lozier et al. 2021) and because elevation can predict *B. terrestris* occurrence (Naeem et al. 2018). Notably, the variables we chose vary substantially across our sampling sites in Tasmania (e.g., Fig. 1, Supp Figs. 1–3 [online only], A1).

Land cover type and vegetation structure have been shown to be ecologically important for bumblebees (Svensson et al. 2000). We, therefore, extracted a range of landcover variables from each site, including ‘% pasture’ (percentage of land used as pasture) from The National Dynamic Land Cover Dataset, Geoscience Australia, Canberra (Lymburner et al. 2010) with a spatial resolution of 250m^2^, derived using data averaged across 2000–2008 (Supp Table 2 [online only], A2). In addition, the variable ‘% open forest and woodland’ was extracted to represent open vegetation since *B. terrestris* prefers foraging and nesting in open rather than closed vegetation (Svensson et al. 2000) (Supp Table 2 [online only], A2). The variable vegetation height (m) (canopy) was extracted as canopy height might be a better predictor of bumblebee species distributions due to flower availability in the understory and/or flowering tree species relevant for bumblebees (Geue and Thomassen 2020) (Supp Table 1 [online only], A2). The variables “% open forest and woodland’ and vegetation height (m) were extracted from the ICESat Vegetation Height and Structure dataset (Scarth 2013) (Supp Table 2 [online only], A2) with a spatial resolution of 30m^2^ and a temporal resolution averaging the years across 2003–2009. *B. terrestris* is also prevalent in urban habitats, such as parks, gardens, and allotment gardens, which it uses for nesting and foraging (Osborne et al. 2008, Lye et al. 2012). The variable ‘% urban area’ was therefore extracted from the Catchment Scale Land Use of Australia dataset (ABARES 2021), capturing data averaged across the years 2008-2019 and with a spatial resolution of 1 km^2^ (Supp Table 2 [online only], A2).

All environmental variables were extracted from raster layers in R by using a circular area of 1 km^2^ around the central coordinates in each sampling site (circle radius from center coordinates = 564 m), to capture the foraging range from the colony reported for *B. terrestris* (Wolf and Moritz 2008). Cell values within the 1 km^2^ area were averaged using weighted mean by cell fraction within the area. The extracted variables were then transformed from cell fraction to percentage of the total 1 km^2^ area.

### Environmental Variable Correlations

To linearize relationships, we logit-transformed the percentage-scale measurements (% urban area, % pasture, and % open forest and woodland), as logit(x)=log[(x+ ε)/(100−x+ε)], where ε is the minimum non-zero percentage value, as recommended when the data include some zeros (Warton and Hui 2011). For the same reason, other environmental variables were natural-log transformed. A Pearson’s correlation matrix R v.1.2.5 (R Core Team 2022) showed that correlations among environmental variables were often high (*r* > 0.70). To avoid collinearity, we therefore only kept a subset of variables correlations *r* < 0.70 for the analyses (Supp Table 2 [online only], A1). The seven environmental variables retained as predictor variables in our analysis included log-transformed measures for MeanAnnualTemp (ºC), MeanAnnualPrecip (mm), SeasonPrecip (mm), vegetation height (m), AvgSummerWind (m/s), and logit-transformed measures for % urban area and % pasture. The variable ‘elevation’, although collinear with other predictors, was used as a single predictor in analyses of wing loading, since a previous study suggested an association between elevation and wing loading (Lozier et al. 2021).

### Morphology × Environment Analysis

All statistical analyses were performed in R v.1.2.5 (R Core Team 2022). Morphological variables were related to environmental predictors using linear mixed-effects models (LMM, lme4), using Site ID as a random effect to control for non-independence of individuals sampled at the same site. The random effect was estimated in all cases, except for analyses of proboscis length, where it was set to 0. In analyses of body weight and proboscis length, both linear and quadratic effects of urban area were included, since a test of linearity indicated the relationships to urbanization were non-linear (Supp Tables 3–4 [online only], A1). To identify important predictors from among the candidates, a ‘full’ LMM model including all candidate predictors was fitted, whereafter insignificant fixed effects were successively pruned using stepwise elimination while respecting marginality (by keeping the linear urbanization variable as long as the quadratic term was in the model). This yielded a ‘final’ model that included only fixed-effect terms with *P*-values ≤ 0.05. *P*-values for fixed effects were calculated by using the R package lmerTest (Kuznetsova et al. 2017) using F-tests with Satterthwaite’s approximation for the denominator degrees of freedom. Conditional statistics were calculated to assess the goodness of fit of the LMM models using the R package modelsummary (Arel-Bundock 2022). To ensure that the model assumptions were met for these and subsequent analyses, we examined diagnostics plots for the models using the R package DHARMA (Hartig 2022).

### Site Relative Abundance

We analyzed site relative abundance using Poisson generalized linear mixed-effects models (GLMM) with a log link using the R package lme4 (Bates et al. 2015), treating the total number of bees captured per site as the dependent variable. To account for variation in sampling intensity among sites, summed sampling time (log-transformed) was used as an offset. The models included Site ID as an observation-level random effect to account for over-dispersion (Harrison 2014). To identify important predictors from among the candidates, a ‘full’ GLMM model was first fitted that included the full set of candidate predictors (listed above) as potential fixed effects. Insignificant fixed effects were successively pruned using likelihood-ratio tests of significance (calculated from chi-squared distributions), yielding a ‘final’ model that included only fixed-effect terms with *P*-values ≤ 0.05.

## Results

### Morphology by Environment Relationships

**Fig. 2.**
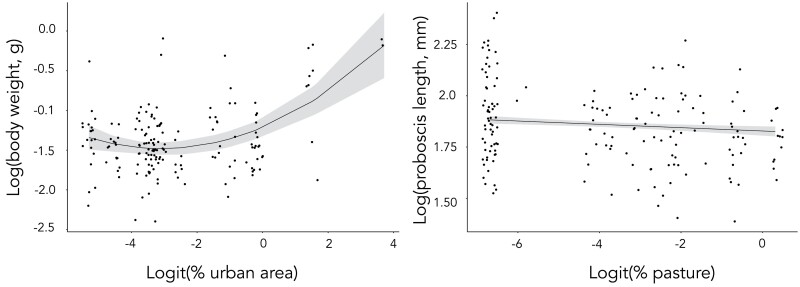
The relationship between a) log body weight and % (logit) urban area (*P* <0.001) with the quadratic relationship shown; b) log proboscis and (logit) % pasture (*P* = 0.0015) with the linear relationship shown. Results are from the final LMM models.

Body weight exhibited a moderate, but significant amount of repeatability (*R* = 0.26, 95% CI: 0.08–0.43, *P* < 0.01), indicating that, while there was more body weight variation within than among sites (*R* < 0.5), average body weight nevertheless varied significantly among sites. The final body weight model obtained using stepwise elimination, indicated that some of this site-to-site variation could be attributed to a relationship with logit-transformed % urban area, as indicated by a significant quadratic term (conditional *R*^2^ = 0.27, *P* < 0.001, Table 2, Fig. 2a). Proboscis length showed moderate but significant repeatability (*R* = 0.08, 95% CI: 0–0.211, *P* = 0.02). Model selection revealed a significant negative relationship with logit-transformed % pasture (conditional *R*^2^ = 0.68, *P* = 0.0015, Table 2, Fig. 2b), but it should be noted that the random effect was estimated as 0 after accounting for variation among sites in % pasture. Proboscis length and body weight had a significant positive relationship (Supp Fig. 4 [online only], A1). Wing loading showed a low but significant repeatability (*R* = 0.08, 95% CI: 0–0.205, *P* = 0.04), and was not significantly correlated with any of the environmental variables considered (conditional *R*^2^ = 0.08, Table 2), including elevation (conditional *R*^2^ = 0.09, *F* = 0.23, *P* = 0.64, Supp Table 5 [online only], A1).

**Table 2.** Summary of Linear mixed model (LMM) fit by REML for the log of body weight, log of proboscis length and the log of wing loading and the log (MeanAnnualTemp, MeanAnnualPrecip, SeasonPrecip, Vegetation height, and Wind speed (in meters per second, m/s) and logit tested environmental variables (% urban and % pasture) using Satterthwaite approximation (F-test). The fixed effect body weight was included in the LMM fit for the log of proboscis length to control for body weight. Significant relationships (*P* =< 0.05) are shown in bold. NumDF = numerator degrees of freedom and DenDF = denominator degrees of freedom are presented. ‘*’ indicates the significant relationship between log proboscis length and logit % pasture, *P* = 0.0015, in the final model. The full model is presented here to show all included environmental variables

Fixed effects	NumDF	DenDF	*F*-value	Pr(>*F*)	NumDF	DenDF	*F*-value	Pr(>*F*)	NumDF	DenDF	*F*-value	Pr(>*F*)
	**Body weight**				**Proboscis length**				**Wing loading**			
**Body weight, g**	–	–	–	–	1	154.27	250.27	**<0.001**	–	–	–	–
MeanAnnualTemp (°C)	1	7.40	0.60	0.46	1	5.70	0.98	0.36	1	8.79	0.11	0.75
MeanAnnualPrecip (mm)	1	7.39	0.09	0.77	1	5.77	0.68	0.44	1	8.72	0.04	0.84
SeasonPrecip (mm)	1	7.93	1.01	0.35	1	6.60	0.06	0.81	1	8.83	0.04	0.84
**% pasture**	1	26.09	1.45	0.24	1	18.35	1.92	*0.18	1	14.65	0.00	0.97
**Poly (% urban, 2)**	2	15.82	8.15	**0.004**	2	13.11	0.84	0.45	–	–	–	–
% urban area	–	–	–	–	–	–	–	–	1	9.57	10.78	0.40
Vegetation height (m)	1	8.01	0.51	0.50	1	6.11	0.08	0.79	1	9.16	0.93	0.36
Wind (m/s)	1	8.28	1.29	0.29	1	6.52	0.93	0.37	1	9.32	0.16	0.70

### Site Relative Abundance of *B. terrestris*

The relative abundance of *B. terrestris* at each site ranged tenfold (from 0.07 to 0.79 bees captured per minute, mean ± S.D.: 0.28 ± 0.18 captured per minute; Table 1). After controlling for sampling effort, site abundance was not related to any of the environmental variables (all *P*-values > 0.09: Table 3).

**Table 3.** Site relative abundance was modeled using a generalized linear mixed-effects model, assuming a Poisson distribution for the dependent variable and a log link function. An offset term, log(time), was included to control for sampling effort. Site ID was included as an observation-level random effect to control for overdispersion. Fixed effects were assessed using likelihood ratio tests of significance. Significant variables (*P* =< 0.05) are shown in bold

Fixed effects	Df	Chisq	Pr (>Chisq)
MeanAnnualTemp (°C)	1	0.49	0.48
MeanAnnualPrecip (mm)	1	1.66	0.20
SeasonPrecip (mm)	1	1.97	0.16
% pasture	1	0.06	0.81
% urban	1	0.00	0.95
Vegetation height (m)	1	1.95	0.16
Wind (m/s)	1	2.87	0.09

## Discussion

We document morphological variation in *B. terrestris* workers across varying environments on the island of Tasmania, three decades after its arrival. On average, the heaviest workers were found in more urbanized sites, possibly due to higher food resource availability. We found a negative association between proboscis length and the percentage of pasture within sites, which may be related to the composition of flower communities, but this was not examined. Wing loading was not related to the environmental conditions within sites. *B. terrestris* was present at all sites across Tasmania with varying relative abundances, but this was unrelated to site environmental variables. What processes drive the observed weak morphological divergence are yet to be further examined, but may reflect either evolutionary change, or perhaps more likely, phenotypic plasticity. Our findings suggest that *B. terrestris* has a strong capacity to thrive in contrasting environmental conditions following a recent invasion.

### Larger Bumblebees Are Associated With Higher Urban Cover

Heaviest *B. terrestris* workers were present in areas with higher urban cover (Fig. 2a), mostly driven by one site (‘Hobart’) that had both the heaviest workers and the highest degree of urbanization (87% urban area) (Supp Fig. 5 [online only], Table 1, A2). Body size in *Bombus* has previously been found to vary with urban areas, but with contrasting results. Theodorou et al. (2020) found *B. terrestris* to be larger in more urbanized areas, whereas Eggenberger et al. (2019) found the opposite pattern for *Bombus pascuorum* (Hymenoptera: *Apidae*) (Scopoli 1763) and *Bombusapidaries* (Hymenoptera: *Apidae*) (Linneaus 1758). Further, Austin et al. (2022) found *Bombus impatiens* (Hymenoptera: *Apidae*) (Cresson 1863) to be larger and *Bombus pensylvanicus* (Hymenoptera: *Apidae*) (De Geer 1773) to be smaller in more urbanized areas.

It can be argued that these differences reflect plastic responses to local conditions (Eggenberger et al. 2019) in urban areas, which can be excellent providers of green spaces, such as parks, domestic and public gardens, allotments, and cemeteries, and provide bees with a wide range of food and nesting resources (Foster et al. 2017, Baldock et al. 2019). However, in other cases, urban areas may limit access to resources because of dominance of impervious areas (e.g., due to concrete/paving), which may result in a loss of foraging resources for bees (Bates et al. 2011). Although the city of Hobart has large garden parks and high floral diversity (pers. observation C. Kardum Hjort), more detailed information about the resource diversity and quality of its urban areas is needed to understand whether the large *B. terrestris* body size observed is related to this.

It has also been argued that body size variation in *Bombus* workers is adaptive (Theodorou et al. 2020). Theodorou et al. (2020) suggested that a positive relationship between worker body size and urbanization was a plastic response to habitat fragmentation in urban areas, since larger bumblebees are more mobile (Greenleaf et al. 2007, Kendall et al. 2022) and potentially able to colonize new habitat fragments. This argument is also consistent with the finding that worker body size in *B. terrestris* is not affected by resource limitation in a laboratory setting (Couvillon et al. 2010). However, the exact processes driving body size variation in Tasmanian *B. terrestris* remains unknown, while genetic and experimental studies are required to help tease apart plastic from adaptive processes. However, our finding of heavier bumblebees in urban areas might indicate increased resource quality and availability, and/or environmental factors associated with the urban environment that favor increased body size.

### Environmental Variation and Proboscis Length

Earlier studies suggest that proboscis length may be a heritable trait that can evolve in response to changing environmental conditions (Miller-Struttmann et al. 2015). After controlling for body weight variation, we found a negative relationship between proboscis length and % pasture (Table 2, Fig. 2), but since the random effect of site ID was estimated as 0, and thus variation in proboscis length within sites was greater than between sites, this result should be interpreted with caution. Pastural areas can be rewarding in terms of floral and nesting resources for bumblebees (Morandin et al. 2007). However, the term ‘pasture’ can represent very different land-cover types, including semi-natural habitat with continuous floral resources, (Noreika et al. 2019), or improved pastures (perennial grasses) with little value for foraging bees (Abdi et al. 2021). In this study, the site with the highest cover of pasture was located in the Northern Midlands of Tasmania, an area dominated by intensive pasture-based agricultural production linked to high intensity cropping of grasses (Lane et al. 2015), which may be of little value to bees. Thus, the land-cover type ‘pasture’ in our study might not describe flower-rich habitat or consists of habitat with fewer floral species. Persson et al. (2015) argued that having a shorter proboscis, compared to varying proboscis length, in intensively farmed landscapes may be due to the bumblebees’ advantage in such a scarcely flowered landscape and where long-tubed flowers are scarce. Thus, they would better match the floral-tube lengths of the cultivated crops.

### No Environmental Effects on Wing Loading

We found no relationship between *B. terrestris* wing loading and environmental variables in Tasmania (Table 2, Supp Table 5 [online only], A1). Lozier et al. (2021) found that the montane bumblebee species, *Bombus vancouverensis cresson* (Hymenoptera: *Apidae*) (Cresson 1878) in the USA exhibited lower wing loading at higher elevations and suggested this to be driven by the effects of high elevation. Low wing loading allows for a more energetically efficient flight in low air densities (Chai and Srygley 1990), an adaptation that may allow bumblebees to survive at high altitudes. However, in the present study, the range of elevations sampled was fairly restricted, not exceeding 660 m a.s.l (Supp Table 1 [online only], A2) which may reduce the need for wing-loading adaptations. Studies investigating intra-specific variation in wing loading in relation to environmental variation are scant, but variation in wing size and shape in relation to landscape type and altitude has been documented in other insects (dragonflies and mosquitoes) (Demirci et al. 2012, Outomuro et al. 2013). The lack of environmental variation in wing-loading in our study may suggest that wing-loading is not a plastic trait affected by landscape variation in Tasmanian bumblebees.

### No Relationship Between Relative Abundance and the Environment

We found no relationship between the relative abundance of *B. terrestris* at a site and corresponding environmental and climatic variables (Table 3). Although the climate and environment varied among sites (Supp Table 1–2 [online only], A2), bumblebees were generally caught in open areas where flowers were present (e.g., along road verges and in patches of grass with flowers). Hence, it is possible that relative abundance is more closely related to small-scale floral resource availability (Westphal et al. 2006, Olsson et al. 2015, Novotny et al. 2021), thus poorly reflecting landscape level abundance (Persson and Smith 2013, Scherber et al. 2019). To truly estimate landscape-wide abundance, stratified sampling across habitats may thus be necessary. In addition, a greater sample size per site may decrease the within-site variance found and help to further elucidate the relationships between site relative abundance and the environmental and climatic variables. The sample size is relatively small per site and possibly limited to capture the full morphological variation.

### Implications for *B. terrestris* Spread to the Australian Mainland

B. *terrestris* was found to be common across the whole extent of Tasmania, demonstrating the species’ capacity to thrive across varying climatic and environmental conditions. The rapid successful expansion of *B. terrestris* across Tasmania is consistent with its invasion success in other countries (Dafni and Shmida 1996, Torretta et al. 2006, Inoue et al. 2008, Morales et al. 2013, Geslin et al. 2017). The morphological variation of *B. terrestris* we found across Tasmania provides evidence for adaptation to variable environments but without strong selection on morphological traits over the past 30 yr. Of concern is that *B. terrestris* already occurs on two off-shore islands (Mewstone and De Witt Island), 6 and 22 km south of Tasmania, respectively (Bryant 1997). This demonstrates the potential for *B. terrestris* to disperse to northern islands and with further island hopping or introduction events, colonize similar environments on the Australian mainland, as previously suggested (Hingston and McQuillan 1998b). In particular, the south and east coastal regions and northwards have been identified as ‘susceptible’ to ‘highly susceptible’ to *B. terrestris* incursions (Acosta et al. 2016) based on climatic niche modeling. This strengthens the argument that invasive *B. terrestris* be considered a significant biosecurity threat to the native ecosystems of New South Wales (NWS), Victoria, and Queensland (QLD) in Australia (as outlined in the Biosecurity Act (2014) Queensland Government (amended 2020), Biodiversity Conservation Act No. 63 (2016), New South Wales Government, and the Flora and Fauna Guarantee Act No. 47 (1998), Victorian Government (amended 2020). Given that the introduction of *B. terrestris* to Tasmania was not accidental (Schmid-Hempel et al. 2007), intentional introduction should also be considered a risk.

Our study identified morphological variation in the introduced *B. terrestris* population across Tasmania that varied according to landscape characteristics (i.e., urban and pasture), which may suggest adaptive processes are occurring that aid dispersal or survival in its invasive range. However, environmental correlations with morphological traits were generally weak, which may instead suggest that this is sufficiently ecologically flexible to be able to invade and exploit a wide range of habitats without having to develop morphological characteristics that are specific to certain environments. Further genetic analyses will help to elucidate further the adaptive processes unfolding within *B. terrestris* following its invasion into Tasmania.

## Supplementary Material

iead006_suppl_Supplementary_Appendix_1

iead006_suppl_Supplementary_Appendix_2
