# Supplementary material for: Morphological Variation in Bumblebees (Bombus terrestris) (Hymenoptera: Apidae) After Three Decades of an Island Invasion
Source: J Insect Sci. 2023 Feb 28;23(1):10. doi: 10.1093/jisesa/iead006 (PMC9972831; doi:10.1093/jisesa/iead006)
Supplement: iead006_suppl_Supplementary_Appendix_1 [file iead006_suppl_supplementary_appendix_1.docx]

**SUPPLEMENTARY MATERIAL**

**Appendix 1**

**Morphological variation in bumblebees (*Bombus terrestris*) (Hymenoptera: *Apidae*) after three decades of an island invasion**

Supplementary tables

**Table S1.** Pearson’s correlation matrix for all naturally logged morphological variables. Highly correlated variables (r > 0.70) were excluded from further analyses. Proboscis length was included as a variable even though it correlated with body length (r= 0.78), by instead correcting for body size variation. The chosen final three morphological variables are shown in bold (body length (mm), proboscis length (mm) and wing loading (g/cm^2^)).

|  | ITD (mm) | **Body weight (g)** | Body length (mm) | **Proboscis length (mm)** | Wing length large wing (mm) | Wing length small wing (mm) | Wing area large wing (mm^2^) | Wing area small wing (mm^2^) | Wing area total (mm^2^) | **Wing loading (g/cm^2^)** |
| --- | --- | --- | --- | --- | --- | --- | --- | --- | --- | --- |
| ITD | 1 |  |  |  |  |  |  |  |  |  |
| **Body weight** | 0.79 | 1 |  |  |  |  |  |  |  |  |
| Body length | 0.59 | 0.79 | 1 |  |  |  |  |  |  |  |
| **Proboscis length** | 0.72 | 0.78 | 0.62 | 1 |  |  |  |  |  |  |
| Wing length large wing | 0.62 | 0.68 | 0.49 | 0.60 | 1 |  |  |  |  |  |
| Wing length small wing | 0.60 | 0.69 | 0.49 | 0.60 | 0.93 | 1 |  |  |  |  |
| Wing area large wing | 0.61 | 0.68 | 0.48 | 0.57 | 0.96 | 0.90 | 1 |  |  |  |
| Wing area small wing | 0.65 | 0.71 | 0.50 | 0.60 | 0.91 | 0.93 | 0.88 | 1 |  |  |
| Wing area total | 0.64 | 0.70 | 0.50 | 0.59 | 0.97 | 0.93 | 0.98 | 0.95 | 1 |  |
| **Wing loading** | 0.22 | 0.34 | 0.36 | 0.23 | -0.28 | -0.22 | -0.30 | -0.21 | -0.28 | 1 |

**Table S2.** Pearson’s correlation matrix for all log (logit % urban, % pasture and % open forest and woodland) environmental variables. Some variables were not used in analyses, to avoid highly correlated variables (r > 0.70) and were removed from further analysis. The chosen final seven environmental variables are shown in bold (MeanAnnualTemp (C°), MeanAnnualPrecip (mm), SeasonPrecip (mm), % pasture, vegetation height (m), % urban area and AvgSummerWind (m/s)).

|  | **MeanAnnualTemp (°C)** | **MeanAnnualPrecip (mm)** | SeasonTemp (°C) | **SeasonPrecip (mm)** | Elevation (m) | **% pasture** | % open forest and woodland | % vegetationcover | **Vegetation height (m)** | **% urban area** | AvgSTempMean (°C) | AvgSTempMax (°C) | AvgSTempMin (°C) | **AvgSummerWind (m/s)** |
| --- | --- | --- | --- | --- | --- | --- | --- | --- | --- | --- | --- | --- | --- | --- |
|  |  |  |  |  |  |  |  |  |  |  |  |  |  |  |
| **MeanAnnualTemp** | 1 |  |  |  |  |  |  |  |  |  |  |  |  |  |
| **MeanAnnualPrecip** | -0.4 | 1 |  |  |  |  |  |  |  |  |  |  |  |  |
| SeasonTemp | -0.54 | -0.05 | 1 |  |  |  |  |  |  |  |  |  |  |  |
| **SeasonPrecip** | 0.02 | 0.47 | -0.11 | 1 |  |  |  |  |  |  |  |  |  |  |
| Elevation | -0.89 | 0.29 | 0.7 | 0.13 | 1 |  |  |  |  |  |  |  |  |  |
| **% pasture** | 0.14 | -0.44 | 0.23 | -0.22 | -0.02 | 1 |  |  |  |  |  |  |  |  |
| % open forest and woodland | 0.03 | -0.38 | 0.6 | -0.12 | 0.26 | 0.7 | 1 |  |  |  |  |  |  |  |
| % vegetation cover | -0.08 | 0.27 | -0.35 | 0.13 | -0.13 | -0.79 | -0.88 | 1 |  |  |  |  |  |  |
| **Vegetation height** | -0.22 | 0.09 | -0.31 | 0 | 0.01 | -0.62 | -0.73 | 0.78 | 1 |  |  |  |  |  |
| **% urban area** | 0.07 | -0.1 | 0.01 | 0.06 | -0.12 | -0.09 | -0.25 | 0.2 | 0.02 | 1 |  |  |  |  |
| AvgSTempMean | 0.96 | -0.44 | -0.36 | -0.01 | -0.84 | 0.26 | 0.14 | -0.16 | -0.3 | 0.13 | 1 |  |  |  |
| AvgSTempMax | 0.66 | -0.38 | 0.23 | -0.03 | -0.43 | 0.39 | 0.53 | -0.4 | -0.59 | 0.11 | 0.78 | 1 |  |  |
| AvgSTempMin | 0.94 | -0.38 | -0.65 | 0.01 | -0.91 | 0.13 | -0.13 | 0.02 | -0.06 | 0.1 | 0.92 | 0.48 | 1 |  |
| **AvgSummerWind** | 0.61 | -0.06 | -0.87 | -0.04 | -0.82 | -0.12 | -0.56 | 0.37 | 0.26 | 0.23 | 0.49 | -0.01 | 0.69 | 1 |

|  | Body Weight | Df | t | Pr(>\|t\|) |
| --- | --- | --- | --- | --- |
| **Fixed effects** | Estimate |  |  |  |
| (Logit) % urban area | 0.17 | 13.27 | 5.05 | **0.001** |
| (Logit) % urban area^2 | 0.04 | 66.02 | 3.37 | **0.002** |
| (Logit) % urban area^3 | 0.00 | 33.56 | 0.92 | 0.363 |

**Table S3.** Log body weight and the significant quadric relationship with logit % urban area (t-test, Satterthwaite's approximation). Significant relationships (P =< 0.05) are shown in bold.

**Table S4.** Log proboscis length and the significant quadric relationship with logit % urban area (t-test, Satterthwaite's approximation). Significant relationships (P =< 0.05) are shown in bold.

|  | Proboscis length | Df | t | Pr(>\|t\|) |
| --- | --- | --- | --- | --- |
| **Fixed effects** | Estimate |  |  |  |
| (Logit) % urban area | 0.06 | 12.66 | 5.56 | **0.0003** |
| (Logit) % urban area^2 | 0.01 | 51.89 | 2.69 | **0.002** |
| (Logit) % urban area^3 | -0.00 | 29.11 | -0.41 | 0.683 |

**Table S5**. Summary over Linear mixed model fit by REML for wing loading and elevation, using Satterthwaite's approximation.

|  |  | | |  |  |
| --- | --- | --- | --- | --- | --- |
| **Fixed effects** | NumDF | DenDF | F-value | Pr(>F) |  |
| Elevation (m) | 1 | 14.151 | 0.23 | 0.64 |  |

Supplementary Figures

**Figure S1.** WorldCLIM variables mean annual temperature (MeanAnnualTemp in °C), temperature seasonality (SeasonTemp in °C), precipitation seasonality (SeasonPrecip in mm) and elevation (m) for Tasmania averaged between the years 1970-2000.

**Figure S2.** Forest cover classes, % vegetation cover and vegetation height for Tasmania from the ICESat Vegetation Height and Structure dataset averaged between the years 2003-2009.

**Figure S3.** WorldCLIM variables wind speed (m s^-1^) in December, January and February for Tasmania averaged between the years 1970-2000.

**Figure S4.** The significant relationship between log proboscis length and log body weight (p <0.001) from the final LMM model. Marginal predictions, 95 % confidence intervals and partial residuals were calculated based on the averages for the logit % pasture variable. Site ID was included in the model to control for non-independence.

**Figure S5**. The relationship between a) log body weight (g) and % (logit) urban area with both a linear and quadratic effect (p <0.001) fitted; b) log proboscis (mm) and (logit) % pasture (p= 0.0015), in both cases using the final LMM models. Marginal predictions, 95 % confidence intervals and partial residuals were calculated based on the averages for the other variables for each model. Overdispersion was handled using site-level random effects. Each sampling site is indicated by an individual colour (see Table 1 for Site ID).
